# Pregnancy-unmasked latent autoimmune arginine vasopressin deficiency with anti-rabphilin-3a antibody positivity

**DOI:** 10.1210/jcemcr/luag291

**Published:** 2026-09-22

**Authors:** Satoshi Yamagata, Mio Naito, Seishi Furukawa, Yoshihisa Sugimura, Seiichiro Higuchi

**Affiliations:** Department of Diabetes, Endocrinology and Metabolism, Kawakita General Hospital, Tokyo 166-8588, Japan; Department of Obstetrics and Gynecology, Kawakita General Hospital, Tokyo 166-8588, Japan; Department of Obstetrics and Gynecology, Kawakita General Hospital, Tokyo 166-8588, Japan; Department of Endocrinology, Diabetes and Metabolism, School of Medicine, Fujita Health University, Aichi 470-1192, Japan; Department of Diabetes, Endocrinology and Metabolism, Kawakita General Hospital, Tokyo 166-8588, Japan

**Keywords:** pregnancy, arginine vasopressin deficiency, anti-rabphilin-3A antibody

## Abstract

Although pregnancy-associated arginine vasopressin (AVP) disorder is rare, it is often attributed to excessive placental vasopressinase activity. However, vasopressinase-independent mechanisms, including gestational unmasking of latent AVP deficiency (AVP-D), may occur. We report a 37-year-old primigravid female who developed severe polydipsia and polyuria in the third trimester. She had hypotonic polyuria with inappropriately low plasma AVP levels and loss of the posterior pituitary bright spot on T1-weighted magnetic resonance imaging (MRI). AVP-D was suspected; desmopressin promptly relieved her symptoms. Desmopressin became unnecessary immediately following delivery; however, at 6 months postpartum, her polydipsia and polyuria recurred. Anterior pituitary dysfunction subsequently developed. Notably, at 6 months postpartum, the posterior pituitary bright spot on MRI remained absent. Herein, the transient postpartum improvement initially mimicked gestational AVP disorder; however, retrospective testing revealed anti-rabphilin-3A antibody positivity, indicating autoimmune hypophysitis involvement. Persistent MRI abnormalities, postdelivery AVP-D recurrence, and subsequent anterior pituitary dysfunction supported an underlying autoimmune etiology. This case raises the possibility that pregnancy may unmask latent autoimmune AVP-D through a vasopressinase-independent mechanism. In patients who develop AVP-D during pregnancy, anti-rabphilin-3A antibody positivity and persistent loss of the posterior pituitary bright spot on MRI may offer crucial diagnostic clues, supporting the significance of long-term endocrine follow-up.

## Introduction

Pregnancy-associated arginine vasopressin (AVP) disorder is rare, with an incidence of 2-4 cases per 100 000 pregnancies [1]. It is most often attributed to increased AVP degradation by placentally derived vasopressinase and may occur through vasopressinase-dependent or -independent mechanisms [2]. Vasopressinase activity increases substantially in late pregnancy and may be enhanced by placental enlargement or impaired hepatic clearance [1-4]. Vasopressinase-dependent disease usually resolves within several weeks postpartum as circulating vasopressinase becomes undetectable [3-5]. However, pregnancy may unmask latent AVP deficiency (AVP-D), in which a previously compensated defect in AVP secretion becomes clinically apparent.

AVP-D, formerly called central diabetes insipidus (DI), results from impaired AVP secretion by the posterior pituitary and may be associated with structural or autoimmune disorders, including lymphocytic infundibulo-neurohypophysitis (LINH) [6, 7]. Loss of the posterior pituitary bright spot on T1-weighted magnetic resonance imaging (MRI) is a typical finding. Anti-rabphilin-3A antibodies are a useful marker of autoimmune LINH [8, 9].

We report a case of AVP-D during pregnancy that initially mimicked gestational AVP disorder because of transient postpartum improvement, but recurred 6 months postpartum and progressed to combined posterior–anterior pituitary dysfunction. Anti-rabphilin-3A antibody positivity suggested an autoimmune mechanism, raising the possibility that pregnancy may unmask latent autoimmune AVP-D through a vasopressinase-independent mechanism.

## Case presentation

A 37-year-old woman (gravida 1, para 0) with no significant medical history was referred to our hospital. Her mother had diabetes mellitus. At 24 weeks and 2 days of gestation, she was diagnosed with gestational diabetes mellitus, which was well controlled with diet alone.

At 29 weeks of gestation, she developed thirst. At 31 weeks and 3 days, she was admitted for marked polydipsia, urinary frequency of ∼10 times daily, vomiting, tremors, dyspnea, and decreased fetal movement. Vital signs were: blood pressure, 117/79 mmHg; heart rate, 109 beats/min; body temperature, 37.1 °C; and oxygen saturation, 98% on room air. Plasma glucose was 93 mg/dL. Cardiotocography showed a baseline fetal heart rate of 160 beats/min with normal variability, transient accelerations, no decelerations, and no uterine contractions.

## Diagnostic assessment

Laboratory evaluation showed no abnormalities, particularly in serum electrolytes (Table 1). Urinalysis was negative for protein and glucose, but urine specific gravity was low at 1.007 (Table 1). After admission, she drank 3-4 L over 12 hours and urinated hourly. AVP-D was suspected, but dynamic endocrine testing was deferred because of concerns about maternal burden during pregnancy.

**Table 1:** Laboratory data on admission and 6 months postpartum

	On admission	6 months	Reference range
Blood biochemistry			
Na	138 mEq/L (138 mmol/L)	140 mEq/L (140 mmol/L)	136−145 mEq/L (136−145 mmol/L)
K	4.2 mEq/L (4.2 mmol/L)	4.2 mEq/L (4.2 mmol/L)	3.5−5.0 mEq/L (3.5−5.0 mmol/L)
Cl	105 mEq/L (105 mmol/L)	99 mEq/L (99 mmol/L)	101−108 mEq/L (101−108 mmol/L)
Osmolality	282 mOsm/kg H_2_O (282 mmol/kg)	280 mOsm/kg H_2_O (280 mmol/kg)	275−295 mOsm/kg H_2_O (275−295 mmol/kg)
Uric acid	3.8 mg/dL (0.23 mmol/L)	4.8 mg/dL (0.29 mmol/L)	3.7−7.8 mg/dL (0.22−0.46 mmol/L)
BUN	3.1 mg/dL (1.1 mmol/L)	8 mg/dL (2.7 mmol/L)	8.0−20 mg/dL (2.9−7.1 mmol/L)
Creatinine	0.46 mg/dL (40.7 μmol/L)	0.76 mg/dL (67.2 μmol/L)	0.65−1.07 mg/dL (57.5−94.6 μmol/L)
Glucose	92 mg/dL (5.1 mmol/L)	90 mg/dL (5.0 mmol/L)	70−109 mg/dL (3.9−6.1 mmol/L)
HbA1c	5.4% (36 mmol/mol)	5.6% (38 mmol/mol)	4.6−5.8 (3.9−6.1 mmol/mol)
Albumin	3.1 g/dL (31 g/L)	—	4.1−5.1 g/dL (41−51 g/L)
AST	29 IU/L (29 U/L)	23 IU/L (23 U/L)	13-33 IU/L (13-33 U/L)
ALT	20 IU/L (20 U/L)	16 IU/L (20 U/L)	6−27 IU/L (6−27 U/L)
γ-GTP	28 IU/L (28 U/L)	9 IU/L (9 U/L)	10−47 IU/L (10−47 U/L)
Alkaline phosphatase	128 IU/L (128 U/L)	59 IU/L (59 U/L)	38−113 IU/L (38−113 U/L)
LDL cholesterol	—	164 mg/dL (4.24 mmol/L)	128−219 mg/dL (3.31-5.66 mmol/L)
HDL cholesterol	—	52 mg/dL (1.4 mmol/L)	40−96 mg/dL (1.0−2.5 mmol/L)
Triglyceride	—	86 mg/dL (0.97 mmol/L)	30−149 mg/dL (0.34−1.68 mmol/L)
Urinary biochemistry			
Na	—	—	130−260 (136−260 mmol/L)
K	—	—	25−50 (25−50 mmol/L)
Cl	—	—	170−250 (170−250 mmol/L)
Osmolality	87 mEq/L (87 mmol/L)	186 mEq/L (186 mmol/L)	50−1300 mEq/L (50−1300 mmol/L)
Hormones			
TSH	1.22 μU/mL (1.22 mIU/L)	2.12 μU/mL (2.12 mIU/L)	0.5−5.0 μU/mL (0.5−5.0 mIU/L)
Free T3	2.26 pg/mL (3.47 pmol/L)	2.44 pg/mL (3.75 pmol/L)	2.3-4.0 pg/mL (3.5-6.2 pmol/L)
Free T4	0.92 ng/dL (11.9 pmol/L)	0.95 ng/dL (12.2 pmol/L)	0.9-1.7 ng/dL (11.6-21.9 pmol/L)
ACTH	49.7 pg/mL (10.9 pmol/L)	8.3 pg/mL (1.83 pmol/L)	7.2-63.3 pg/mL (1.6-13.9 pmol/L)
Cortisol	31.5 μg/dL (869 nmol/L)	5.5 μg/dL (152 nmol/L)	6.2-19.4 μg/dL (171-535 nmol/L)
DHEAS	—	652 ng/mL (1.77 µmol/L)	230-2660 ng/mL (0.62-7.21 µmol/L)
AVP	0.7 pg/mL (0.65 pmol/L)	1.1 pg/mL (1.01 pmol/L)	0-4 pg/mL (0-3.7 pmol/L)
PAC	—	2.97 ng/dL (82 pmol/L)	0.4-8.2 ng/dL (11-227 pmol/L)
PRA	—	0.8 ng/mL/h	0.2−2.3 ng/mL/h
LH	0.3 mIU/mL (0.3 IU/L)	3.9 mIU/mL (3.9 IU/L)	1.8-10.2 mIU/mL (1.8-10.2 IU/L)
FSH	<0.1 mIU/mL (<0.1 IU/L)	10.6 mIU/mL (10.6 IU/L)	3.0-14.7 mIU/mL (3.0-14.7 IU/L)
Estradiol	20 000 pg/mL (73 400 pmol/L)	<10 pg/mL (<37 pmol/L)	<47 pg/mL (<173 pmol/L)
Progesterone	—	—	<0.33 ng/mL (<1.05 nmol/L)
PRL	166.2 ng/mL (166.2 µg/L)	9.3 ng/mL (9.3 µg/L)	4.9-29.3 ng/mL (4.9-29.3 µg/L)
GH	25.2 ng/mL (25.2 µg/L)	3.8 ng/mL (3.8 µg/L)	0.13-9.88 ng/mL (0.13-9.88 µg/L)
IGF-1	263 ng/mL (34.4 nmol/L)	165 ng/mL (21.6 nmol/L)	70-201 ng/mL (9.2-26.3 nmol/L)

Values in parenthesis are International System of Units (SI).

Abbreviations: ACTH, adrenocorticotropic hormone; ALT, alanine aminotransferase; AST, aspartate aminotransferase; AVP, arginine vasopressin; BUN, blood urea nitrogen; DHEAS, dehydroepiandrosterone sulfate; FSH, follicle-stimulating hormone; GH, growth hormone; HbA1c, hemoglobin A1c; HDL, high-density lipoprotein; IGF-1, insulin-like growth factor-1; LDL, low-density lipoprotein; LH, luteinizing hormone; PAC, plasma aldosterone concentration; PRA, plasma renin activity; PRL, prolactin; TSH, thyroid-stimulating hormone; γ-GTP, gamma-glutamyl transpeptidase.

**Figure 1:**
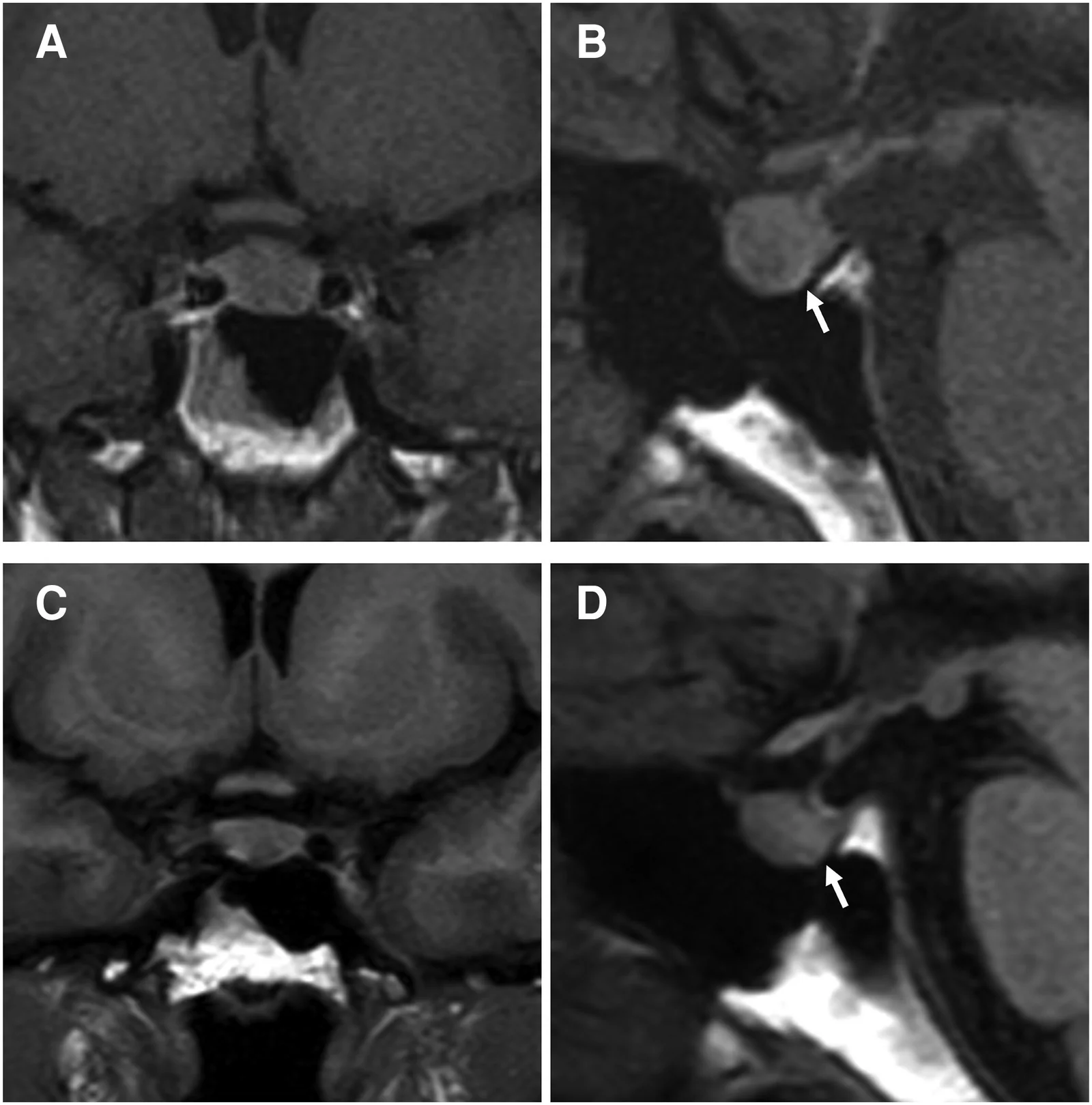
Pituitary magnetic resonance imaging (MRI). (A, B) MRI performed during pregnancy revealing loss of the normal posterior pituitary bright spot (white arrows) with pituitary enlargement (9 mm). (C, D) MRI performed at 6 months postpartum showing improvement in pituitary enlargement (6.5 mm); however, the posterior pituitary bright spot remained absent (white arrows).

**Figure 2:**
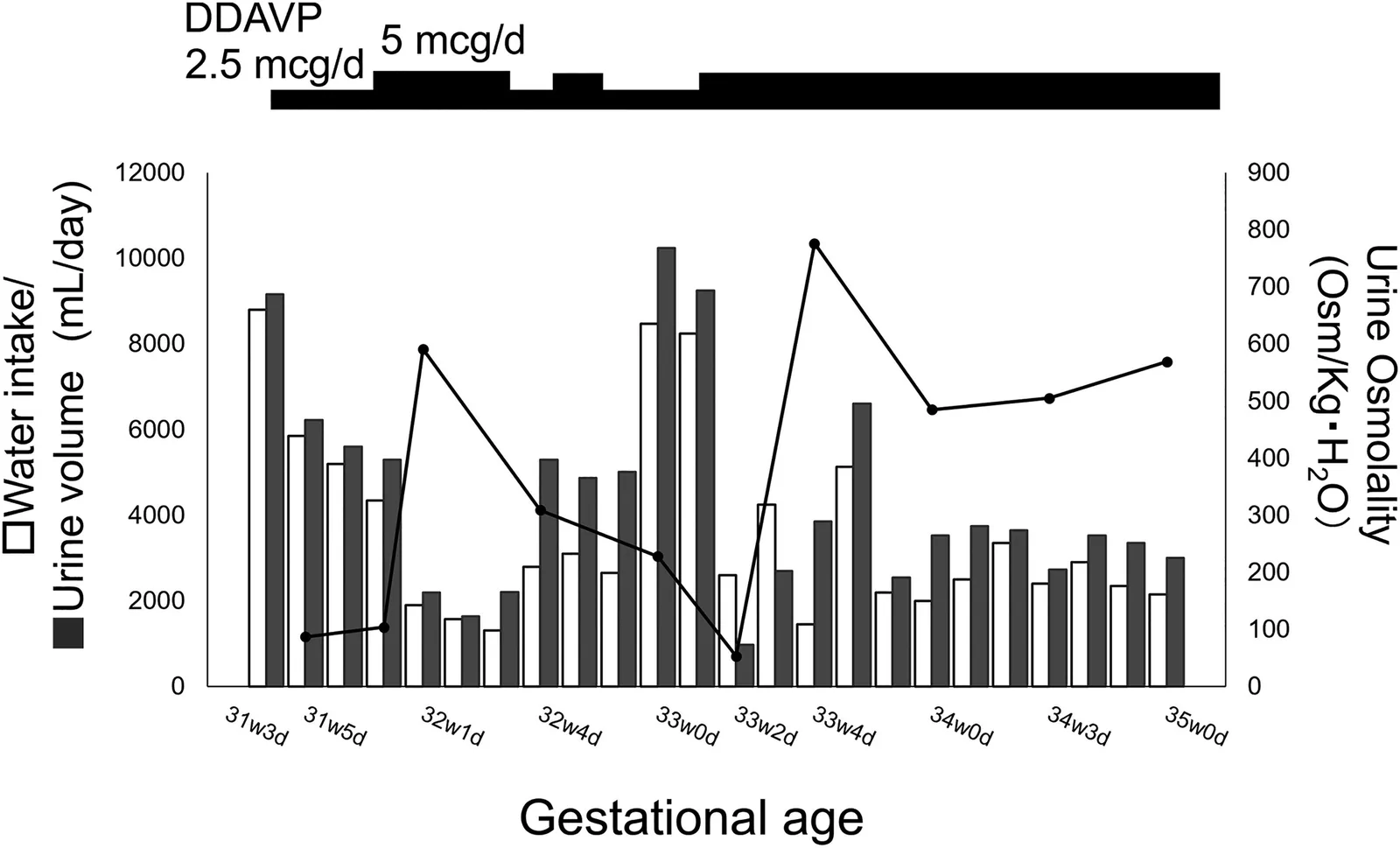
Changes in water intake, urine volume, and urine osmolality during deamino D-arginine vasopressin therapy. Open bars indicate water intake, closed bars denote urine volume, and the solid line represents urine osmolality.

Urine osmolality was 87 mOsm/kgH_2_O [SI: 87 mmol/kg] (reference range, 50-1300 mOsm/kgH_2_O [SI: 50-1300 mmol/kg]); plasma AVP was inappropriately low at 0.7 pg/mL [SI: 0.65 pmol/L] (reference range, 0-4 pg/mL [SI: 0-3.7 pmol/L]) despite plasma osmolality of 282 mOsm/kgH_2_O [SI: 282 mmol/kg] (reference range, 285-290 mOsm/kgH_2_O [SI: 285-290 mmol/kg]). Anterior pituitary hormone levels were within pregnancy-specific normal ranges (Table 1). Pituitary MRI showed mild enlargement (9 mm) and loss of the posterior pituitary bright spot on T1-weighted images (Fig. 1A and 1B). AVP-D was suspected, and intranasal deamino D-arginine vasopressin (DDAVP) 2.5 mcg/day was initiated. At 5 mcg/day, water intake and urine output stabilized at ∼3 L/day, with serum sodium, serum osmolality, and urine osmolality of 131 mEq/L (reference range, 136-145 mEq/L [SI: 136-145 mmol/L]), 270 mOsm/kgH_2_O [SI: 270 mmol/kg], and 500 mOsm/kgH_2_O [SI: 500 mmol/kg], respectively. She was discharged at 35 weeks of gestation (Fig. 2).

At 38 weeks and 2 days, she underwent spontaneous vaginal delivery of a female infant weighing 2796 g, with 1- and 9-min Apgar scores of 8 and 9 and umbilical artery pH of 7.32. Placental weight was 560 g. DDAVP was discontinued on postpartum day 1 without an increase in water intake or urine output. At 8 weeks postpartum, thirst, polyuria, and polydipsia had completely resolved, and urine osmolality was 322 mOsm/kgH_2_O [SI: 322 mmol/kg].

At 6 months postpartum, polyuria (15 times/day) and polydipsia (3-4 L/day) recurred. Urine osmolality, serum sodium, and plasma AVP were 186 mOsm/kg [SI: 186 mmol/kg], 140 mEq/L [SI: 140 mmol/L], and 1.1 pg/mL [SI: 1.01 pmol/L], respectively (Table 1). IgG4 was <5 mg/dL [SI: <0.05 g/L] (reference range, 11-121 mg/dL [SI: 0.11-1.21 g/L]). DDAVP 2.5 mcg/day was reinitiated. Follow-up MRI showed persistent loss of the posterior pituitary bright spot (Fig. 1C and 1D). Retrospective testing of serum obtained at 20 weeks of gestation revealed anti-rabphilin-3A antibody positivity. In the 5% hypertonic saline infusion test, AVP failed to increase despite elevated serum sodium (Table 2), supporting AVP-D with posterior pituitary involvement and suggesting an autoimmune etiology. At 6 months postpartum, adrenocorticotropic hormone (ACTH) and cortisol levels at 9:00 Am were low (8.3 pg/mL [SI: 1.83 pmol/L] and 5.5 µg/dL [SI: 152 nmol/L], respectively) (reference ranges, 7.2-63.3 pg/mL [SI: 1.6-13.9 pmol/L] and 6.2-19.4 µg/dL [SI: 171-535 nmol/L]), as was estradiol (E2) (<10 pg/mL [SI: <37 pmol/L]; reference range, <47 pg/mL [SI: <173 pmol/L]) (Table 1). She also reported fatigue, but no apparent loss of body hair; lactation after delivery was normal. Further endocrine testing showed low basal and peak cortisol levels on corticotropin-releasing hormone (CRH) stimulation (5.7 and 16.8 µg/dL [SI: 157 and 463 nmol/L], respectively), despite an ACTH increase from 14.0 to 78.7 pg/mL [SI: 3.1 to 17.3 pmol/L] (Table 3). On luteinizing hormone-releasing hormone (LH–RH) stimulation, LH and follicle-stimulating hormone (FSH) increased appropriately, and E2 was restored to 39.9 pg/mL [SI: 146 pmol/L] at 7 months postpartum. Thyroid-stimulating hormone and prolactin responses were preserved. Peak cortisol on rapid ACTH stimulation was 15.2 µg/dL [SI: 419 nmol/L] (Table 3), supporting adrenal insufficiency [10]. Hydrocortisone 15 mg/day was initiated. MRI at 6 months postpartum showed persistent loss of the posterior pituitary bright spot despite regression of pituitary enlargement to 6.5 mm (Fig. 1C and 1D).

**Table 2:** Result of the hypertonic saline infusion test

Time (min)	0	30	60	90	120
Na	139 mEq/L (139 mmol/L)	139 mEq/L (139 mmol/L)	141 mEq/L (141 mmol/L)	143 mEq/L (143 mmol/L)	144 mEq/L (144 mmol/L)
AVP	0.9 pg/mL (0.83 pmol/L)	0.9 pg/mL (0.83 pmol/L)	0.9 pg/mL (0.83 pmol/L)	0.8 pg/mL (0.74 pmol/L)	0.8 pg/mL (0.74 pmol/L)

The 5% hypertonic saline infusion test was performed at 6 months postpartum. Values in parenthesis are International System of Units (SI).

Abbreviation: AVP, arginine vasopressin.

**Table 3:** Endocrinological examinations

CRH + LH–RH + TRH test				
Time (min）	0	30	60	90
ACTH	14 pg/mL (3.1 pmol/L)	78.7 pg/mL (17.3 pmol/L)	62.9 pg/mL (13.8 pmol/L)	39.3 pg/mL (8.7 pmol/L)
Cortisol	5.7 µg/dL (157 nmol/L)	14.6 µg/dL (402 nmol/L)	16.8 µg/dL (463 nmol/L)	13.5 µg/dL (372 nmol/L)
LH	8.0 mIU/mL (8.0 IU/L)	71.5 mIU/mL (71.5 IU/L)	79.6 mIU/mL (79.6 IU/L)	80.3 mIU/mL (80.3 IU/L)
FSH	7.2 mIU/mL (7.2 IU/L)	10.1 mIU/mL (10.1 IU/L)	12.2 mIU/mL (12.2 IU/L)	12.8 mIU/mL (12.8 IU/L)
TSH	1.9 μU/mL (1.9 mIU/L)	18 μU/mL (18 mIU/L)	15.1 μU/mL (15.1 mIU/L)	10.7 μU/mL (10.7 mIU/L)
PRL	13 ng/mL (13 µg/L)	181 ng/mL (181 µg/L)	105 ng/mL (105 µg/L)	61.5 ng/mL (61.5 µg/L)
Rapid ACTH test				
Time (min)	0	30	60	
Cortisol	7.8 µg/dL (215 nmol/L)	13.2 µg/dL (364 nmol/L)	15.2 µg/dL (419 nmol/L)	
Aldosterone	6.98 ng/dL (194 pmol/L)	15.3 ng/dL (425 pmol/L)	13.8 ng/mL (382 pmol/L)	

Human CRH (100 mcg), LH-RH (100 mcg), TRH (200 mcg), and ACTH (250 mcg; Synacthen) were administered intravenously. Values in parenthesis are International System of Units (SI).

Abbreviations: ACTH, adrenocorticotropic hormone; CRH, corticotropin-releasing hormone; FSH, follicle-stimulating hormone; LH, luteinizing hormone; PRL, prolactin; TSH, thyroid-stimulating hormone.

## Treatment

Intranasal DDAVP was initiated at 2.5 mcg/day during pregnancy and increased to 5 mcg/day, stabilizing water intake and urine output at ∼3 L/day and normalizing urine osmolality to approximately 500 mOsm/kgH_2_O [SI: 500 mmol/kg]. DDAVP was discontinued on postpartum day 1 after symptom resolution and restarted at 2.5 mcg/day when polyuria and polydipsia recurred 6 months postpartum. Hydrocortisone 15 mg/day was initiated after adrenal insufficiency was confirmed, resulting in complete resolution of fatigue. No glucocorticoid-related adverse events occurred.

## Outcome and follow-up

The patient delivered a healthy female infant at 38 weeks and 2 days by spontaneous vaginal delivery. Thirst and polyuria resolved postpartum without DDAVP, and urine osmolality normalized by 8 weeks postpartum.

At 6 months postpartum, AVP-D recurred, requiring DDAVP. Adrenal insufficiency was also diagnosed. MRI showed persistent loss of the posterior pituitary bright spot with regression of pituitary enlargement. DDAVP and hydrocortisone were continued without unexpected adverse events.

## Discussion

The transient postpartum resolution of polydipsia and polyuria initially mimicked gestational AVP disorder. Pregnancy-related physiological changes may have temporarily concealed AVP-D [11]. Vasopressinase-dependent AVP disorder is generally transient because vasopressinase becomes undetectable by ∼5-6 weeks postpartum [1, 2]. Multiple gestations, placental enlargement, and hepatic dysfunction can increase the risk [3-5]. Our patient had neither multiple gestations nor hepatic dysfunction, and the placental weight was within the normal range [4]. In gestational AVP disorder, the posterior pituitary bright spot is often preserved on T1-weighted MRI [4], whereas it remained absent in our patient at 6 months postpartum. However, MRI findings are not specific for AVP-D, as the bright spot persists in 20% of patients with complete and 40% with partial central AVP-D [7]. Anti-rabphilin-3A antibodies provided additional evidence of impaired AVP secretion [8]. Although copeptin is a useful biomarker, particularly after hypertonic saline stimulation [12], it was not measured because it is not widely available in Japan. Instead, AVP-D was supported by low plasma AVP, an inadequate response to 5% hypertonic saline, persistent loss of the posterior pituitary bright spot, and a response to DDAVP. These findings argue against vasopressinase-dependent AVP disorder.

Besides AVP, oxytocin can also be degraded by vasopressinase because of structural similarity [13]. During pregnancy, increased placental oxytocinase activity enhances oxytocin clearance, while physiological function is maintained through coordinated regulation of secretion, receptor expression, and signaling [14]. Thus, placental aminopeptidase activity affects both AVP and oxytocin metabolism during pregnancy, with potentially different physiological consequences.

The pituitary gland physiologically enlarges during pregnancy and regresses after delivery [15]. Although the MRI findings at 6 months postpartum were not typical of autoimmune hypophysitis, the reduced pituitary height was consistent with physiological regression. Nevertheless, anti-rabphilin-3A antibody positivity suggested an autoimmune contribution to AVP-D [8]. Rabphilin-3A is expressed in the posterior pituitary and hypothalamic vasopressin neurons [8]. Anti-rabphilin-3A antibodies were detected in 76% of clinically diagnosed LINH cases and 100% of biopsy-proven cases [8]. A recent study reported sensitivities of 100% and 80% for LINH and lymphocytic panhypophysitis, respectively, with 97.4% specificity for distinguishing these disorders from other sellar or suprasellar lesions [16]. Although anti-rabphilin-3A antibody-positive AVP-D due to LINH has been reported during late pregnancy, those cases did not show combined anterior–posterior pituitary dysfunction [4, 17, 18].

During gestation, placental CRH stimulates the maternal pituitary–adrenal axis, approximately doubling plasma ACTH levels [19]. CRH secretion is transiently suppressed at 3 and 6 weeks postpartum and normalizes by 12 weeks [19]. In our patient, adrenal insufficiency was diagnosed at 6 months postpartum despite a preserved ACTH response to CRH stimulation. The pregnancy-related increase in ACTH may have masked underlying adrenal insufficiency during gestation. The suboptimal cortisol response to direct adrenal stimulation with rapid ACTH further suggested reduced adrenal reserve. Although elevated prolactin during pregnancy and lactation physiologically suppresses LH and FSH secretion [20], low estradiol at 6 months postpartum with normalized prolactin suggested hypothalamic or pituitary dysfunction rather than physiological suppression. Impaired corticotropic and gonadotropic function with preserved thyrotropic and lactotrophic function supported partial pituitary dysfunction. Despite the absence of pituitary biopsy, the clinical findings and anti-rabphilin-3A antibody positivity strongly support an immune-mediated mechanism.

In conclusion, AVP-D was supported by polyuria and polydipsia, low plasma AVP, an inadequate response to hypertonic saline, persistent loss of the posterior pituitary bright spot on MRI, and a prompt response to DDAVP. Anti-rabphilin-3A antibody positivity further supported an autoimmune mechanism underlying combined posterior and anterior pituitary dysfunction. Because AVP-D may transiently improve, long-term follow-up is essential to detect recurrence.

## Learning points

- Autoimmune AVP-D can present during pregnancy and transiently improve postpartum, closely mimicking gestational AVP disorder.
- The absence of the posterior pituitary bright spot on T1-weighted MRI and anti-rabphilin-3A antibody positivity may help differentiate AVP-D from gestational AVP disorder.
- Anti-rabphilin-3A antibody positivity may be associated with both posterior and anterior pituitary dysfunction, highlighting the need for long-term endocrine follow-up.

## Data Availability

Original data generated and analyzed for this case report are included in this published article.
